# Assessment of Pozzolanic Activity of Ground Scoria Rocks under Low- and High-Pressure (Autoclave) Steam Curing

**DOI:** 10.3390/ma15134666

**Published:** 2022-07-03

**Authors:** Galal Fares, Abdulrahman M. Alhozaimy

**Affiliations:** Center of Excellence for Concrete Research and Testing, College of Engineering, King Saud University, P.O. Box 800, Riyadh 11421, Saudi Arabia; alhozimy@ksu.edu.sa

**Keywords:** scoria rocks, pozzolanicity, single-chain and 3D-silicate minerals, low-pressure steam curing, autoclave curing, nano-tobermorite formation

## Abstract

Two sources of natural scoria rocks were procured and ground for use in concrete as natural pozzolans (NP1 and NP2). The evaluation of their pozzolanic reactivity is carried out using different techniques and approaches. The primary goal of employing these techniques is to monitor the amount of portlandite (CH=Ca(OH)_2_) consumed during steam curing at low or high pressure. The pozzolanicity of NP powders is determined either directly by monitoring CH variation or indirectly by compressive strength and microstructure development. Autoclave curing is known to stimulate the pozzolanicity of the inert siliceous and aluminosiliceous materials under its high-pressure steam conditions. Both steam-curing conditions were applied in this investigation. In this study, X-ray diffraction, scanning electron microscope, thermogravimetric, Fourier transform infrared, and isothermal analyzers were used. It is concluded that the nature and types of minerals in SR determine their pozzolanic reactivity as either low-pressure steam-reactive or high-pressure steam-reactive cementitious materials. Due to the nature of their silicate structures, notably single-chain or 3D-framework structures, plagioclase feldspars (albite-anorthite) minerals are high-pressure steam-reactive minerals, whereas pyroxene (enstatite and diopside) minerals are low-pressure steam-reactive minerals. Using high-pressure steam curing, varied replacement levels of up to 60% were achieved in NP1, with a consistent strength activity index (SAI) of 99%, while an SAI of 79% was obtained with NP2. During low-pressure steam curing, NP1 and NP2 consumed around 72 and 80% of portlandite, respectively, demonstrating their relative pozzolanic reactivity. When compared to the control concrete mix, the strength activity indices of NP1, NP2, and class F fly ash in their normal concrete mixes reached 74.3, 82, and 73.7%, respectively, after 56 days of normal curing conditions.

## 1. Introduction

The Arabian Peninsula’s volcanic scoria cones have been shown to be a cost-effective source of scoria rocks (SRs) that have yet to be fully explored. These types of rocks are located in an area of around 90,000 km^2^, making it one of the world’s largest areas, extending along the Red Sea on the Arabian Peninsula’s western side [1]. SRs are widely spread in the Arabian Peninsula and have been used as lightweight fine and coarse aggregates [2,3]. The potential of the utilization of the ground SR in concrete as natural Pozzolan has been investigated by many researchers [4,5,6,7,8]. Since ancient Greek and Roman times, natural pozzolanic materials have been utilized as building materials. The concrete construction sector benefits from the usage of natural Pozzolan on a technical, economic, and environmental level. Pozzolanic materials are characterized with amorphous aluminosilicate phase. The amorphous phase, as the reactive phase, in the presence of moisture, namely, low-pressure steam curing, forms a cementitious matrix through a pozzolanic reaction with hydrated lime (calcium hydroxide). The pozzolanic reactivity of pozzolanic materials differs from one source to another due to variation in their physicochemical properties [6]. As a result, the quantity of reactive phase in the pozzolanic material participating in the pozzolanic reaction should be evaluated. The accurate amount of pozzolanic material to be added to consume the calcium hydroxide (CH) liberated during the hydration of Portland cement (PC) can be established by estimating the ratio of reactive and inert silicates in pozzolanic materials [7]. Diatomite, trass, bentonite, and zeolite were used as natural pozzolanic materials with varying pozzolanic reactivity due to their variable amorphous-phase content and specific surface area [9]. Taftan natural pozzolan was combined with inorganic activators such as sodium hydroxide and sodium silicate to develop an inorganic polymeric binder [10]. Several studies on conventional and nonconventional wastes as pozzolanic materials have been published to show the pozzolanic properties from different perspectives [11,12]. However, when compared to other methods, there are fewer studies that cover autoclave curing of cementitious materials (Alawad et al., 2015 [13]). For example, dune sand was ground and used as a cement replacement material that becomes extremely active during autoclave curing [14]. The authors have shown in a previous study that autoclave curing of a mix of ground dune sand, a highly crystalline silica, and hydrated lime in a defined stoichiometric ratio of 1.32 resulted in an optimized autoclaved construction unit [15]. Furthermore, due to the incorporation of 30% ground crystalline sand, nano-C-S-H structures, identified as the tobermorite phase, with a characteristic Ca/Si ratio of 1.09, were observed [15]. These structures have led to a 2.5 h compressive strength equivalent to a 28-day compressive strength under normal curing and absence of ground silica. The higher 2.5 h autoclave compressive strength of the mix with ground crystalline silica, which is equivalent to the 28-day compressive strength developed under normal curing and in the absence of powdered silica, was due to these newly formed nanostructures. No such study covering the autoclave curing of scoria rocks as natural pozzolanic materials could be found.

The higher iron content of SR is hypothesized to function as a barrier, slowing pozzolanic reactivity [1]. The quantity of reactive phases may also be enhanced by applying an accelerated curing technique. One of these procedures, autoclave curing, or high-pressure steam curing, allows for the rapid production of precast concrete for a variety of immediate applications [16,17,18,19]. The enhanced properties are attributed to the improved microstructure and decreased pore system with maximum refinement [20]. Precast and normal concrete technology will benefit from this study. From the literature, it is concluded that scoria rocks are categorized as pozzolanic materials that exhibit a wide range of pozzolanic reactivity without explaining underlying reasons. The contribution of this study, on the other hand, demonstrates that the major minerals found in scoria rocks can be divided into single-chain or 3D silicate structures that can be activated using the appropriate technique depending on the dominant microstructure. The best technique for 3D silicate structures is the use of high-pressure steam-curing conditions while the single-chained silicate is activated under normal low-steam-curing conditions. Therefore, the main objectives of the study are to evaluate the pozzolanic reactivity of SR from two sources and compare it to class F fly ash and ground inert silica using both mechanical and chemical methods, namely, autoclave and low-pressure steam curing. The two basic methods for evaluating pozzolanic reactivity were the development of compressive strength under autoclave curing and the monitoring of variations in Portlandite content under steam curing. One of the techniques for stimulating the pozzolanic reactivity is energy-consuming (autoclave curing), and the other is low-energy-consuming (steam curing) compared to autoclave curing. Then, this study ends up with a validation of the results in normal concrete mix compared to class F fly ash and control concrete mixes. The behavior of SR powders under each technique of activation was evaluated. Both techniques were discussed, compared, and assessed in this study. The pozzolanic reaction of SRs with calcium hydroxide was investigated using X-ray diffraction (XRD), Fourier transform infrared (FTIR), simultaneous thermogravimetric analysis–differential scanning calorimetry (TGA-DSC) and isothermal microcalorimetric techniques.

## 2. Experimental Work

### 2.1. Materials

**Figure 1:**
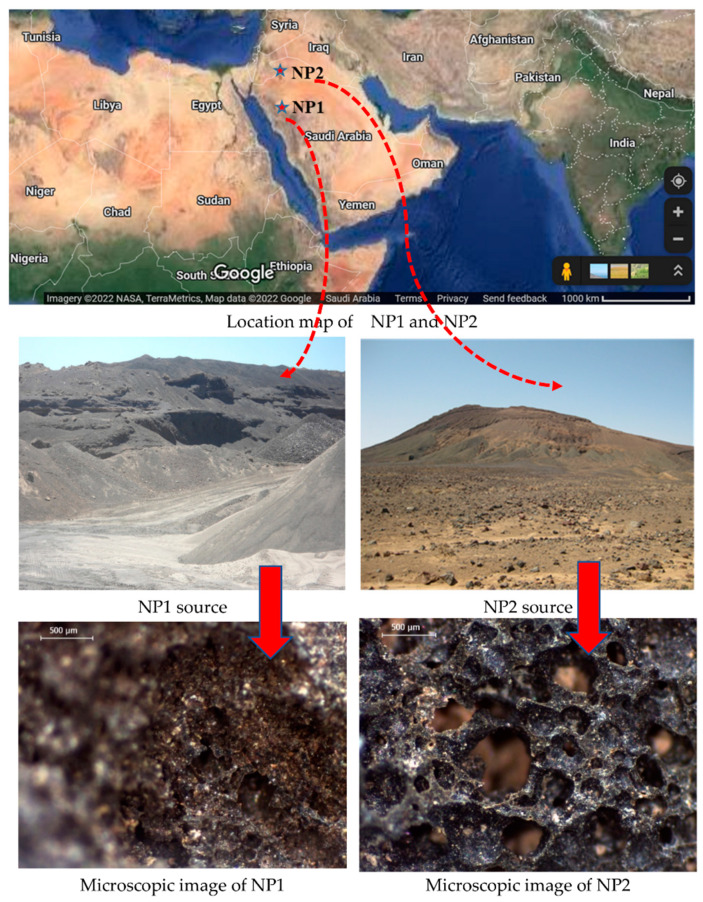
Optical microscopic investigation of typical NP1 and NP2 grains before grinding; location map is an actual satellite illustration of the two NP sources in the Kingdom of Saudi Arabia (Map data ©2022 Google).

Ordinary Portland cement (PC) complying with ASTM C150 was used as the main binder to which other powders were added as a partial cement replacement. Class F fly ash (as per ASTM C618) and ground crystalline silica flour (GSF) were used for comparison and assessment. GFS is composed mainly of 99.5% silica. As natural pozzolans, two samples of scoria rocks from the Arabian Peninsula’s western region were employed in this investigation (NP1 and NP2), as shown in Figure 1. The NP1 and NP2 samples were pulverized in a ball-mill machine until about 90% of the powder passed through a sieve with a 75 µm opening size. The particle size distributions of the powder samples were measured using a laser particle-size distribution analyzer (LA 950V2, Horiba). For concrete mixtures, an optimum mix of dune and crushed sands (65% DS and 35% CS, respectively) was used with crushed aggregates of nominal grain sizes of 10 mm and 20 mm (30% CA10 and 70% CA20, respectively). The saturated surface dry (SSD) densities of DS, CS, CA10, and CA20 were 2.65, 2.65, 2.63, and 2.64, respectively. X-ray fluorescence (XRF) was used to determine the chemical compositions of powder samples (Model Axios from PANalytical). The SEM analysis was conducted using a Versa 3D scanning electron microscope (SEM from FEI), while X-ray diffraction (XRD) analysis was performed on an Empyrean diffractometer from PANalytical. XRD analysis was conducted with an adjusted scan speed of 2 deg/min and a Cu Ka radiation generator at 40 kV and 30 mA. The Fourier transform infrared (FTIR tensor 27 from Bruker) spectroscopic analysis of fine powders was conducted using a standard KBr method in the transmission mode at 20 °C, 32 scans with a spectral resolution of 4 cm^−1^. The vibration region in the range of 4000–400 cm^−1^ was used to record the FTIR spectra. The variation in Ca(OH)_2_ content was measured using a simultaneous TGA-DSC apparatus (Model TA, Q600 SDT) in the presence of nitrogen gas (50 mL/min) at a heating rate of 20 °C/min. TGA-DSC was also used to calibrate the Ca(OH)_2_ dosage in the investigated samples and evaluate the pozzolanic reactivity. The heat flow and heat of hydration of cement pastes containing NP1, NP2, GFS and FA were measured using isothermal conduction calorimetry. The tests were conducted on model TAM Air isothermal calorimeter of 8 channels for a period of about 80 h and operating temperature of 25 °C.

### 2.2. Mixing Procedure

Different paste mixtures with different replacement levels of NP1 and NP2 were prepared. The replacement levels vary from 10% to 90%, with an increment of 10%. Due to the enhancing autoclaving effect on GSF, as reported by the authors [15], different ternary mixtures of PC and GFS with NP of the lowest autoclave pozzolanic activity were further investigated. The main goal of testing these mixtures was to figure out the best method of GSF incorporation in the binder, namely as an addition or replacement of NP. With 75% NP and 25% hydrated lime, the pozzolanicity of NP mixtures was also monitored and measured. A W/B ratio of 0.5 was applied to all mixes.

### 2.3. Curing Regimes

Two curing regimes were applied in this study. Autoclave curing (high-pressure steam curing) was used with cement-paste samples. The mixtures prepared with hydrated lime were low-pressure steam cured in an environmental chamber of adjusted temperature and relative humidity.

#### 2.3.1. Autoclave Curing

The cement pastes were prepared as per ASTM C305 and cured for 24 h under standard curing conditions of temperature (20 ± 2 °C) and relative humidity of 100%. The samples were then moved to a rack in the autoclave chamber, beneath which a liter of water was added. The temperature was then raised from ambient to 160 °C during a 60-min period, allowing the accumulated pressure to reach 5 bars. After that, the pressure was raised to 10 bars by raising the chamber temperature to 180 °C for 60 min. Following this, the heaters were switched off and the fans were turned on to bring the temperature to the desired level in roughly an hour.

#### 2.3.2. Steam Curing

For the steam curing of hydrated lime samples, an environmental chamber with controlled temperature and humidity was utilized as a reactor to accelerate the pozzolanic reactivity under the effect of temperature and humidity. The temperature was adjusted at 80 °C with an elevated relative humidity of 100% where samples were kept for 7 days, after which they were moved to a drier at 80 °C to remove free water in about 24 h under a closed system to avoid carbonation.

### 2.4. Testing Procedure

The binary and ternary cementitious mixes were prepared according to ASTM C305. The dry and wet mixes, which were examined immediately after mixing, provided calibration data for thermal analysis. SEM and FTIR were employed to further analyze the samples obtained utilizing both curing procedures.

## 3. Results and Discussion

### 3.1. Materials Characterization

**Figure 2:**
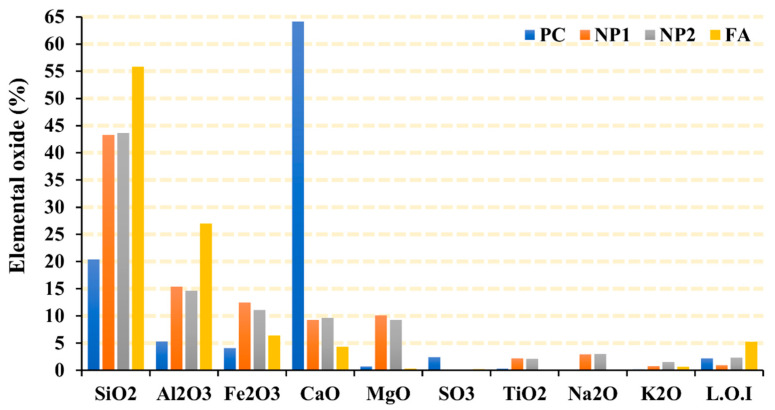
Chemical composition of PC and powders.

**Figure 3:**
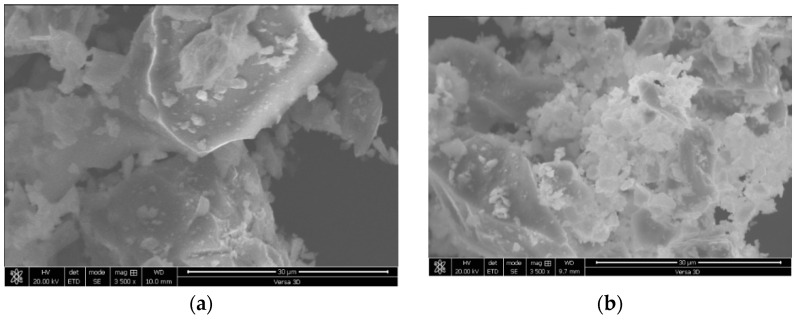

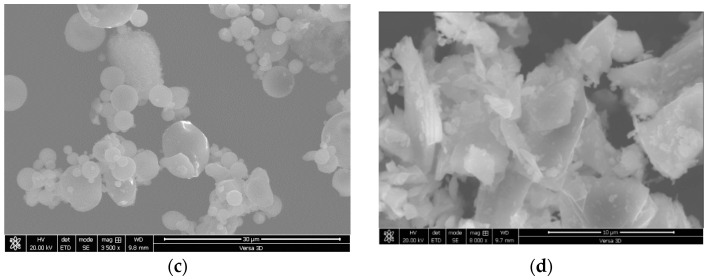
SEM images of (**a**) NP1, (**b**) NP2, (**c**) FA, and (**d**) GSF.

**Figure 4:**
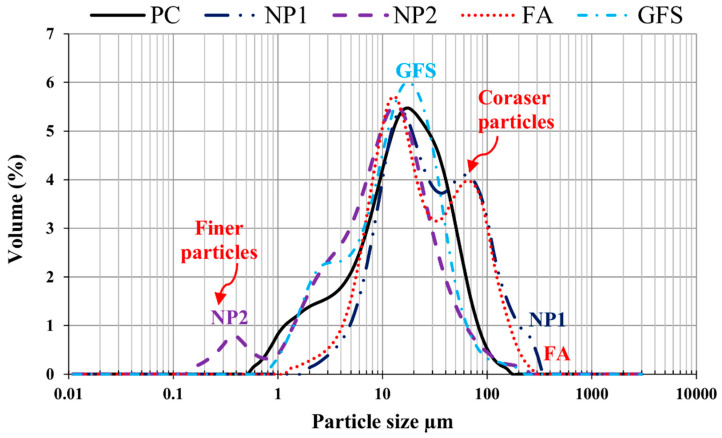
Volume-based particle-size distribution curves of PC, NP1, NP2, FA, and GSF.

The chemical analysis of fine powder is shown in Figure 2. Both NP1 and NP2 have similar chemical compositions. The main elements found in cement and cementitious materials are found in both NP samples. The sum of the percentages of the main oxides (SiO_2_, Al_2_O_3_, and Fe_2_O_3_) exceeds the recommended sum of 70% by ATSM C618, which makes NP suitable for use as natural pozzolan compared to fly ash. The chemical analysis has revealed that the only difference in the chemical composition is that NP1 contains more magnesium than NP2 and that both have the same elements found in cement and fly ash. The SEM photomicrographs and XRD analysis of natural pozzolans (NP1 and NP2), FA, and GSF are shown in Figure 3 and Figure 4, respectively.

**Figure 5:**
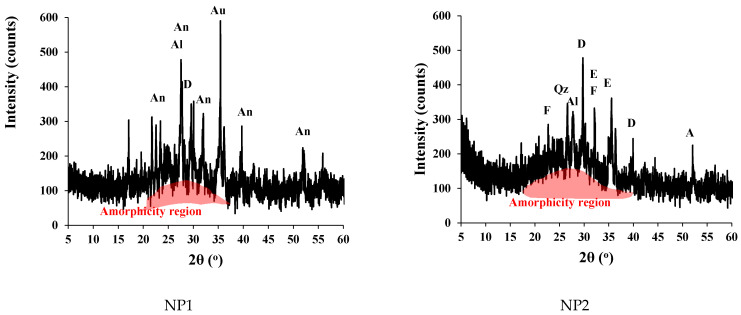

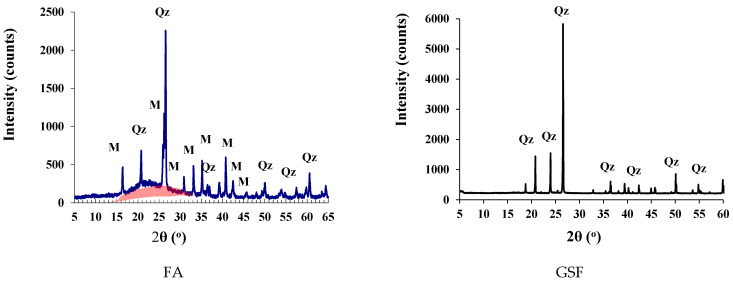
XRD analysis of fine powders. Al: albite, An: anorthite, Au: augite, E: enstatite, D: diopside, F: frosterite, M; mullite Qz: quartz.

**Figure 6:**
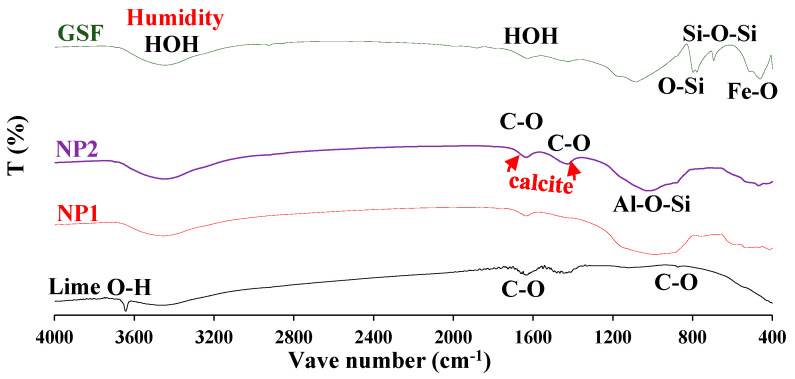
FT−IR of lime, NP1, NP2, and GSF powders.

**Figure 7:**
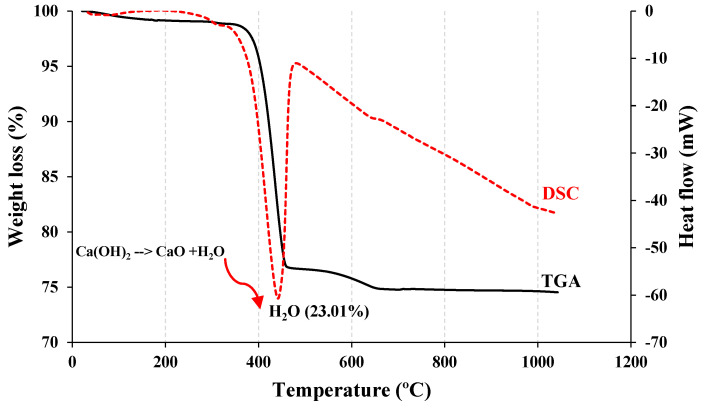
TGA and DSC analyses of hydrated lime.

The particle-size distribution analysis shows that NP1 is coarser than NP2 and other powders. NP2 and FA have similar particle distributions. The median particle diameters of PC, NP1, NP2, FA, and GFS are 14, 18, 12, 20, and 13 µm, respectively. The scanning electron photomicrographs of powders confirm that NP1 is coarser than NP2. The angular structures of PC, NP1, NP2, and GFS due to the grinding process are apparent. The presence of cenosphere particles in FA powder has been confirmed. The morphological structures of fine powders reveal the comparable fineness as inferred from Figure 4. The XRD analysis has confirmed that there is a wide variation in the mineralogical compositions of the NP1 and NP2 samples, as shown in Figure 5. NP1 is composed mainly of plagioclase feldspars of the albite-anorthite group of minerals, while NP2 is composed of enstatite and diopside, which belong to the minerals of the pyroxene family. Feldspars are known for their 3D-framework structures characteristic of tectosilicate. On the other hand, pyroxene minerals are known to have single-chain silicate structures specific to inosilicate minerals. A GSF with strong quartz peaks was chosen as a highly crystalline and inert material. In order to investigate the tested powder and simulate pozzolanic reactivity, FA was chosen as an amorphous pozzolanic material to be catalyzed by Portlandite (Ca(OH)_2_). XRD analysis shows that NP2 is more amorphous than NP1. Plagioclase feldspars such as albite (sodium aluminosilicate (NaAlSi_3_O_8_)), anorthite (calcium aluminosilicate (CaAl_2_Si_2_O_8_)) and augite (calcium sodium magnesium iron aluminosilicate (Na, Ca) (Mg, Fe, Al)(Al, Si)_2_O_6_) are found as the major minerals in NP1. On the other hand, pyroxene minerals such as enstatite (magnesium silicate (MgSiO_3_)) and diopside (calcium and magnesium silicate (CaMgSi_2_O_6_)) are found as the major minerals in NP2. Albite-anorthite groups of minerals are known as unstable minerals under hydrothermal conditions, which dissociate in the presence of Portlandite into CASH, while diopside and enstatite are more stable under such conditions [1]. The reaction of albite with lime under autoclave conditions is reported to lead to the formation of CASH gel compositions (wairakite), while that of quartz leads to the formation of tobermorite [20]. FTIR analysis in Figure 6 shows the presence of different absorption bands at 3441 cm^−1^, O-H stretching, and 1640 cm^−1^ for O-H bending, attributed to the humidity in the sample. The absorption band at 1427 cm^−1^ is for the asymmetric stretching mode of calcite. The absorption band at 455 cm^−1^ is related to O-C-O bending vibration. The amount of H_2_O in pure Ca(OH)_2_ is theoretically calculated to be 24.3%. Ca(OH)_2_ with a purity of 95% contains approximately 23.08% H_2_O. According to these findings, Ca(OH)_2_ is contaminated with calcite, and a portion of it is carbonated. Therefore, this explains its purity of 95%. NP2 is contaminated with calcite minerals. These data agree with the chemical analysis shown in Figure 2. As a result, the estimated amount of H_2_O in Ca(OH)_2_ is approximately 23.01%, as shown in Figure 7.

### 3.2. High-Pressure Steam (Autoclave) Curing

**Figure 8:**
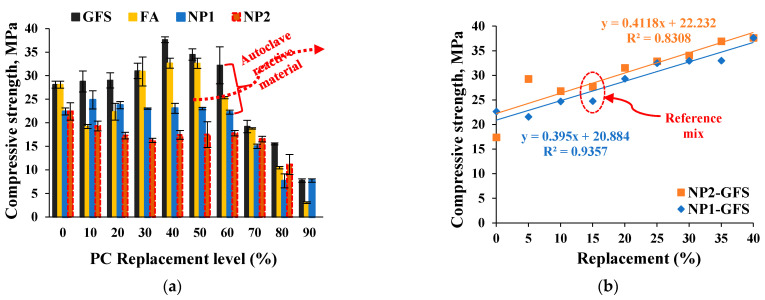
Effects of (**a**) GFS, FA, NP1, and NP2 replacement level on strength development and (**b**) replacing up to 40% NP1 and 40% NP2 by GSF on improving compressive strength.

**Figure 9:**
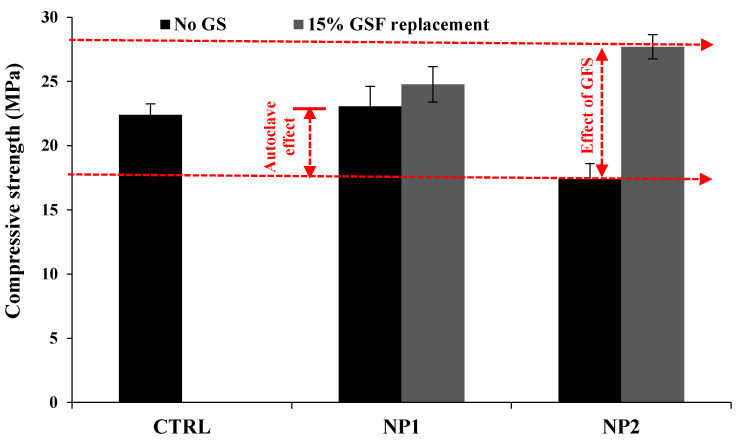
Performance of 25% NP1 and NP2 with 15% ground silica flour.

Autoclave curing of cementitious systems is a quicker tool for stimulating pozzolanic activity of siliceous materials and accelerating the process of cement hydration. The effect of the replacement level of NP in cement paste on NP1 and NP2 is shown in Figure 8. The autoclave curing has shown that NP1 contains autoclave-active ingredients. These ingredients of plagioclase feldspar minerals that are identified in NP1 are reported to show autoclave pozzolanic activity in contrast to the pyroxene minerals of NP2. The enhancing effect of NP1 replacement levels on the compressive strength under autoclave curing is evident until a replacement level of 60%, as shown in Figure 8a. On the other hand, the deteriorating effect of the NP2 replacement level on the development of compressive strength is noted until 30% NP1 is reached. However, it is noted that the replacement level of 40% NP1 until 60% NP1 leads to an improvement in compressive strength. NP2 recovers its strength at a replacement level of 40%. Accordingly, the replacement level of 40% can be considered the optimal content for both NP1 and NP2. In this regard, the autoclave pozzolanic reactivity of GSF is well-reported to enhance the strength of its pastes [15]. Accordingly, the effect of GSF replacement level on the optimal content of 40% NP1 was investigated. The incorporation of GFS leads to an additional enhancement in the overall pozzolanic reactivity of the mix, as shown in Figure 8b. The effect of partially replacing the optimal NP content (40%) by 15% GFS (15% GFS + 25% NP2) on the development of compressive strength is shown in Figure 9. In contrast to the weaker 3D-framework phases in NP1, the interaction of single-chain silicate phases in NP2 with crystalline silica results in stronger hydration products of high strength. As a result, pyroxene (single-chain silicate) minerals containing ground crystalline silica are strongly advised for use in autoclave curing. These findings will benefit both precast and normal concrete technology. Precast concrete is useful for rapid strength development, with 1 day being equivalent to 28 days, as well as having a strong construction unit with negligible shrinkage. When compared to time-consuming traditional construction practices, it allows for faster structure installation, which means less time and less cost. The latent pozzolanic properties of NP under normal curing could be overcome using autoclave curing, allowing SRs to be used in more construction applications. The construction unit with SR powders becomes more resistant to thermal degradation due to their volcanic origin.

#### 3.2.1. Microstructural Analysis

**Figure 10:**
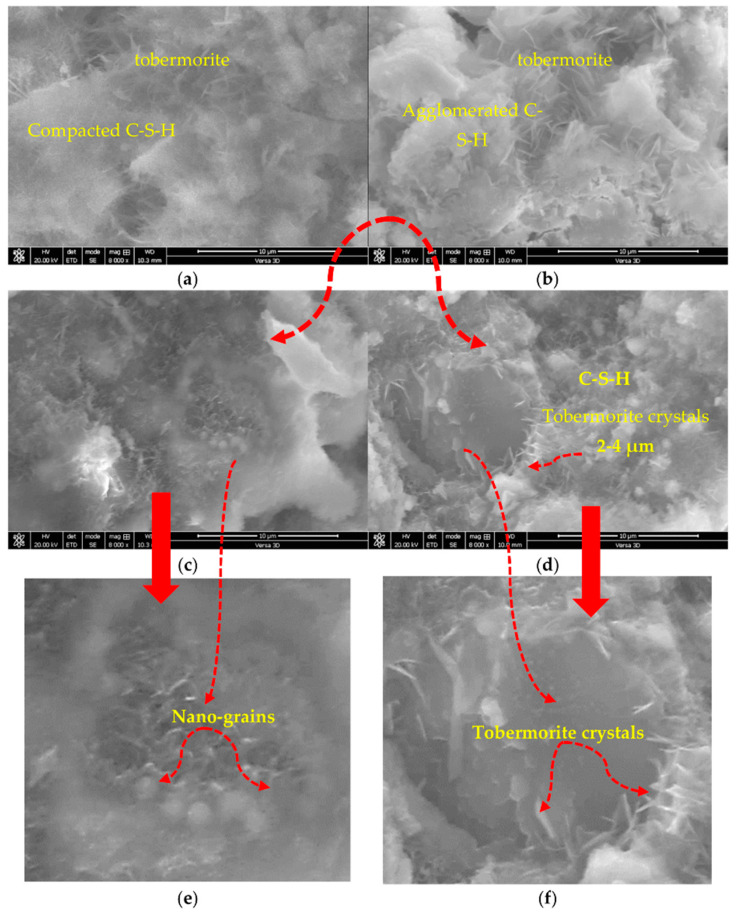
SEM images of (**a**) 40% NP1, (**b**) 40% NP2, (**c**)15%GFS25%NP1, (**d**) 15%GFS25%NP2 and their additional magnifications as in (**e**) and (**f**), respectively.

The microstructural analysis of paste mixtures has revealed the presence of two types of C-S-H structures, namely compacted and agglomerated, as shown in the NP1 and NP2 mixtures, respectively. As shown in Figure 10a,b, the compacted C-S-H (amorphous phase) in NP1 is interlaced with tobermorite crystals (crystalline form of C-S-H), whereas pure tobermorite formation with nanogranules of C-S-H is evident in NP2 (2–4 µm in size). Tobermorite is a highly crystalline form of C-S-H, whereas C-S-H is amorphous or quasi-crystalline in nature. The incorporation of GFS has confirmed the reported findings with NP1 and NP2. The replacement level of 15% GFS with 25% NP1 has led to an obvious integration between C-S-H formed by NP1 and the additional tobermorite due to the presence of GFS, as confirmed in Figure 10c. Furthermore, the presence of nanosized structures in the NP1 mix was confirmed, and the improvement in compressive strength was attributed to this type of microstructural integration between C-S-H and tobermorite. Due to the obvious structure of tobermorite crystals, the increased amount of evidently produced tobermorite in the (25% NP2 and 15% GFS) mix greatly contributed to its strength, as shown in Figure 10d.

#### 3.2.2. Reaction Visualization

**Figure 11:**
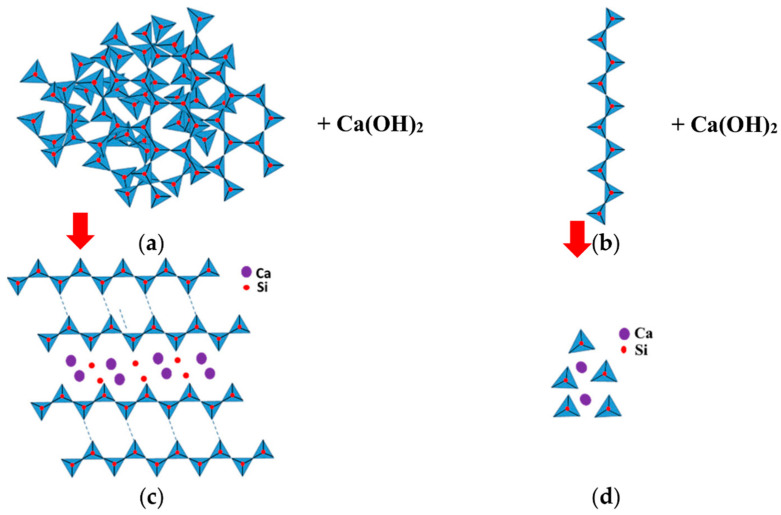
Schematic representation of the autoclave reaction products of Portlandite and different forms of (**a**) 3D-framework tectosilicates; (**b**) single-chain inosilicates compounds; and (**c**,**d**) their reaction products, respectively, as simply visualized by the authors.

Figure 11 depicts the high-pressure steam-curing interaction of NP minerals, both single-chain and 3D-framewok silicate compounds, based on silicate-group literature [21,22,23,24]. The simplified high-pressure steam-curing reactions show that the reaction of tectosilicate complex structures with hydrated lime leads to tobermorite formation. The reaction of single-chain inosilicates with hydrated lime, on the other hand, is expected to result in the depolymerization of single silica tetrahedra and the creation of porous noncondensed C-S-H, as demonstrated in Figure 11.

### 3.3. Low-Pressure Steam Curing

This section of the investigation presents various analyses of NP1 and NP2 mixes with Ca(OH)_2_ that were treated under low-steam-curing conditions. After the curing stage, samples were divided into two groups: those produced through steam curing (SC) and those obtained through drying (Dry-SC).

#### 3.3.1. Thermogravimetric Analyses

**Figure 12:**
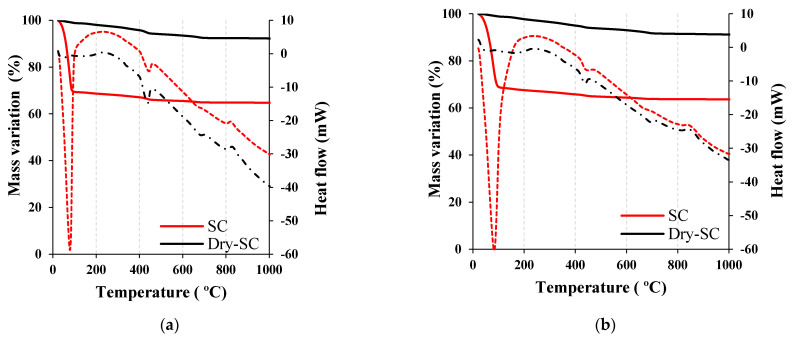
Thermal analysis of (**a**) NP1−lime mixture and (**b**) NP2−lime mixture.

The thermal analyses of NP1 and NP2 samples are shown in Figure 12a,b, respectively. From the figure, it is evident that the samples as obtained (SC) contain free-water content of about 30.62 and 31.1% that is maximally liberated at about 79 and 84 °C from NP1 and NP2 mixes, respectively. The estimated amounts of the remaining CH in the NP1 and NP2 are about 7.1 and 5.1%, respectively. This implies that during the pozzolanic reaction, NP1 and NP2 consumed approximately 72 and 80% of portlandite, respectively. Accordingly, the pozzolanic reactivity of NP2 under low-steam curing is higher than that of NP1. At this point, it can be concluded that NP2 pyroxene minerals (single-chain inosilicates) carry pozzolanic properties under normal conditions, while NP1 plagioclase feldspars (3D-framework tectosilicates) do not. The autoclave pozzolanic properties, specifically, stimulated pozzolanic properties under high-pressure steam curing, result from the decomposition and depolymerization taking place under the alkaline environment triggered by CH to generate different forms of tobermorite, which might be explained by these 3D structures.

#### 3.3.2. FT-IR Analysis

**Figure 13:**
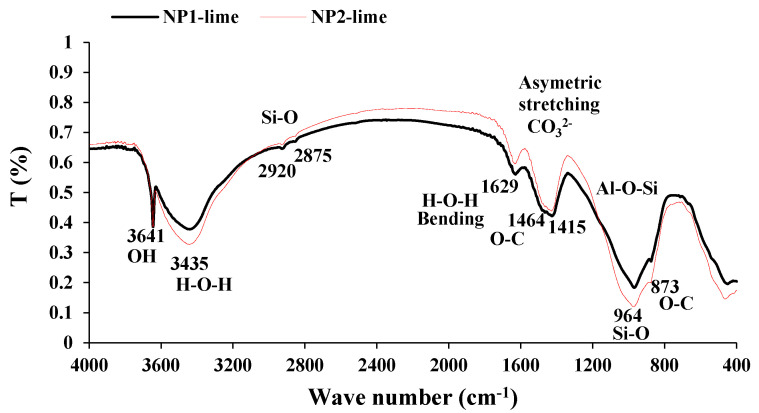
FT−IR spectrum for NP1−lime and NP2−lime mixes.

The FT-IR analysis results are shown in Figure 13. The hydroxyl group in Ca(OH)_2_ causes the sharp and narrow absorption band at 3641 cm^−1^. The absorption band at 964 cm^−1^ due to Si-O stretching is attributed to the formed C-S-H [25]. This absorption band broadens with the formation of C-S-H, as observed in NP2-lime mix compared to NP1-lime mix. The complex structures of tectosilicates found in NP1 make the reaction under low-pressure steam curing slow while it is accelerated with the single-chain inosilicates found in NP2. These findings confirm the same information obtained from thermal analysis.

#### 3.3.3. XRD Analysis

**Figure 14:**
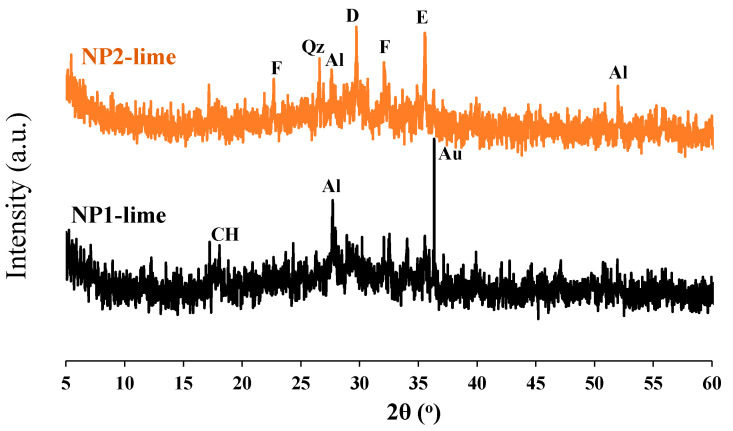
XRD analysis of tested NP–lime mixes. Al: albite, An: anorthite, Au: augite, E: enstatite, D: diopside, F: frosterite, M; mullite Qz: quartz; CH: portlandite.

The mineralogical variation of the NP1 and NP2 mixes reacted with lime under low-pressure steam curing is shown in Figure 14. From the figure, it is noted that the amount of CH remaining in the NP1 mix with lime is within the detection limit of the XRD machine, in contrast to the NP2 mix with lime, which shows traces of CH. When comparing Figure 5 with Figure 14, it can be inferred that some of the diopside and enstatite minerals disappeared in the NP2 mix with lime. As a result, it is concluded that these minerals are the main phases that participate in the pozzolanic reaction.

### 3.4. Validation of the Findings

To validate the findings in this work, 25% NP1 and 25% NP2 performances were tested in normal concrete (NC) mixes compared to control mix as well as mixes containing 25% replacement level of class F fly ash (FA), as a known pozzolanic material, and ground silica flower (GSF), as an inert material. The mix composition with a water-to-binder ratio of 0.5 and NP1, NP2, FA, and GFS replacement level of 25% was used, as detailed in Table 1. The benchmark mix was the concrete mix with 25% GFS, which is considered an inert material. To evaluate the pozzolanicity of NP1 and NP2, the concrete mix with FA is used as the reference pozzolanic mix.

#### 3.4.1. Fresh Properties

**Figure 15:**
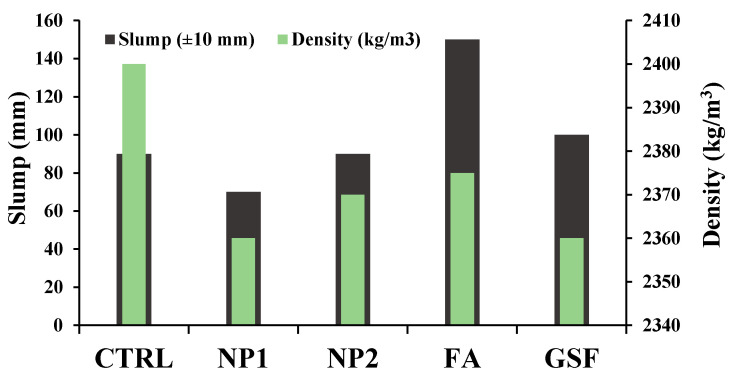
Fresh properties of concrete.

The results of the slump and density of concrete mixes with NP1, NP2, FA, and GFS in addition to the control (CTRL) mix are presented in Figure 15. The results have shown insignificant variation in the slump values for concrete mixes incorporating NP1, NP2, and GFS compared to the control mix. However, the incorporation of FA has significantly increased the slump. The enhancement in slump of FA mix is well-understood and has been attributed to the spherical form of its particles, while the similar effect of other powders on the slump has been attributed to the angular structure of their particles due to grinding during preparation, as demonstrated in Figure 2. Regarding the density of fresh concrete mixes, a slight difference between the control and blended mixes was observed, as presented in Figure 15.

#### 3.4.2. Hardened Properties

**Figure 16:**
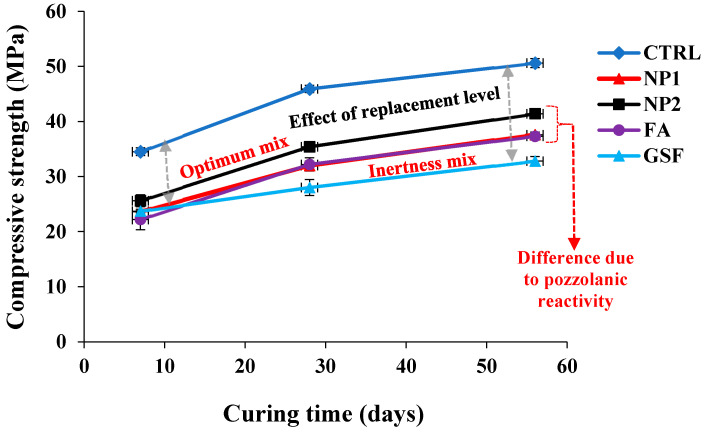
Comparison of compressive-strength development of normal concrete mixes containing NP1, NP2, FA, and GSF.

The results of concrete mixes are shown in Figure 16. The inertness lines of the compressive strength developed by GFS at 28 and 56 days are defined, as shown in Figure 16. At 28 and 56 days, both FA and NP1 have shown similar trends. The latent pozzolanic properties of FA are shown until a curing age of 56 days. On the other hand, at 28 and 56 days, NP2 had pozzolanic properties that exceeded both FA and GFS, with strength activity indices of 77 and 82%, respectively. The effect of replacement level is defined by the distance between the control and GFS mixes. The NP2 mix falls in the middle of this range, indicating that it has a higher pozzolanic reactivity than NP1 and FA.

#### 3.4.3. Temperature Profiles

**Figure 17:**
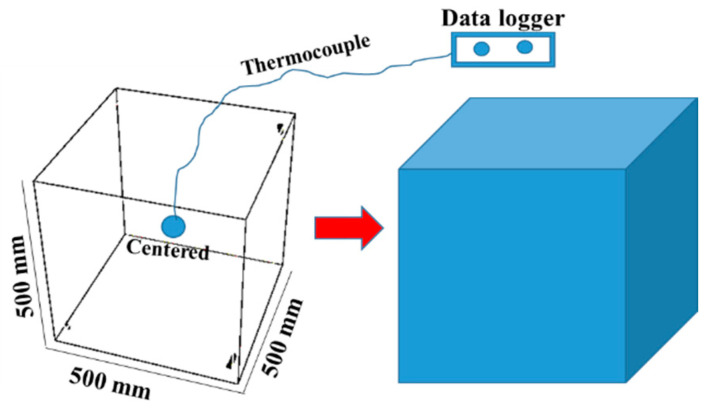
Temperature profiles measurement using custom-made semiadiabatic system.

There are several experimental methods to predict the temperature rise in concrete [26]. The temperature increases over time of concrete mixes, namely the temperature profile, is monitored in each mix using a custom-made semiadiabatic test setup shown in Figure 17. A semiadiabatic technique was adopted in this study due to its simplicity [27,28]. The molds were prepared using plywood formworks over-lined with 16 mm-thick polystyrene sheets, in which the concrete final sizes reached 500 × 500 × 500 mm^3^, as depicted in Figure 17. Thermocouple wires connected to a data logger are installed in the middle of the cubic molds to monitor the temperature profiles in a time interval of about 3 days. At this point, the temperature becomes parallel to the ambient temperature line.

**Figure 18:**
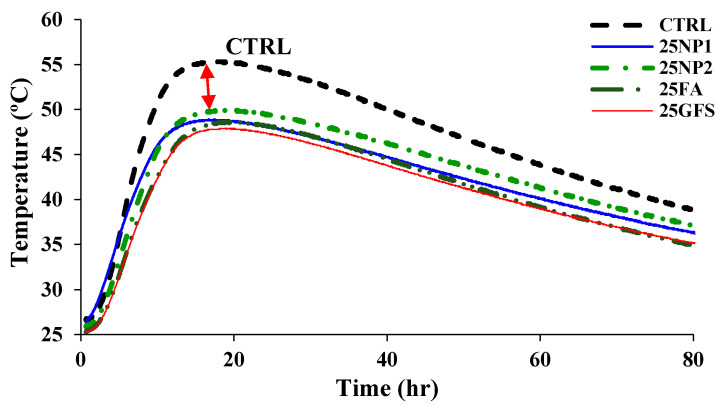
Concrete core temperature of concrete mixes compared to control mix (CTRL).

Figure 18 shows the measured temperature profiles. The temperature reached its maximum peak value in the control mix (CTRL), followed by the NP2 mix with a notable temperature gap. Then, NP2, FA, and GFS followed NP2, where GFS was the mix with the minimum peak value. The replacement level of 25% was the main reason for the temperature gap between the CTRL curve and other powders’ curves, especially at the peak values, as identified by the red arrow. The pozzolanic reactivity of NP2 contributed to the increase in temperature due to its reactivity with cement grains. The early latent pozzolanic properties of FA were the main reason for its known temperature-reducing behavior. The lowest peak was attributed to the inertness of the crystalline quartz of GFS. The difference between the maximum point on the CTRL peak and the GFS peak is about 13.5% (with a difference of about 7.5 °C).

#### 3.4.4. Heat Flow and Heat of Hydration

**Figure 19:**
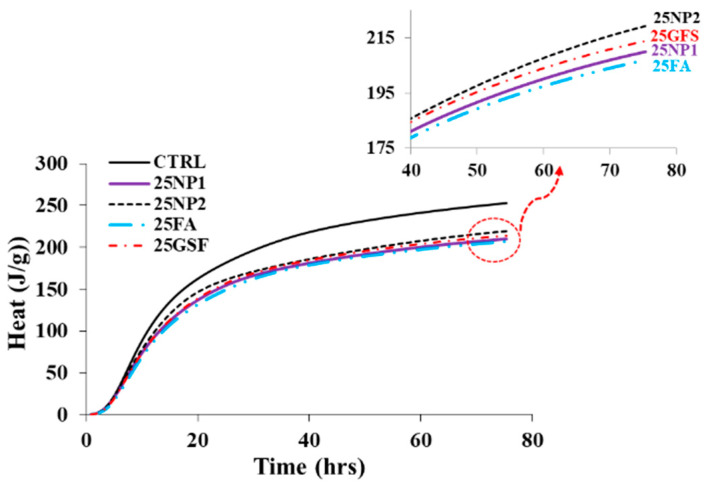
Isothermal analyses of concrete mixes showing total heat of hydration until 80 h.

The heat flow and total heat of hydration of the same pastes of the concrete mixes are shown in Figure 19. The control mix shows the maximum heat flow followed by the NP2 mix, followed by GFS, NP1, and FA. As mentioned in PSD analysis, the median particle diameters of PC, NP1, NP2, FA, and GFS are 14, 18, 12, 20, and 13 µm, respectively. It is evident that the heat flow is highly affected by the median diameter of the powders incorporated with cement, known as the filler effects, which act as nucleation sites for cement particles [29,30,31]. The total heat of hydration accumulated until a curing age of about 80 h was 252.6 J/g for the control mix, while it reached 219 J/g for the NP2 mix, followed by GFS, NP1, and FA mixes of total heat of hydration of 214, 210, and 206 J/g, respectively. From the isothermal analysis, it is evident that the heat flow is highly affected by the median diameter of the powders incorporated into cement, with finer sizes accelerating hydration, known as the filler effects, which act as nucleation sites for cement particles, as shown in Figure 19.

#### 3.4.5. Microstructural Analysis

**Figure 20:**
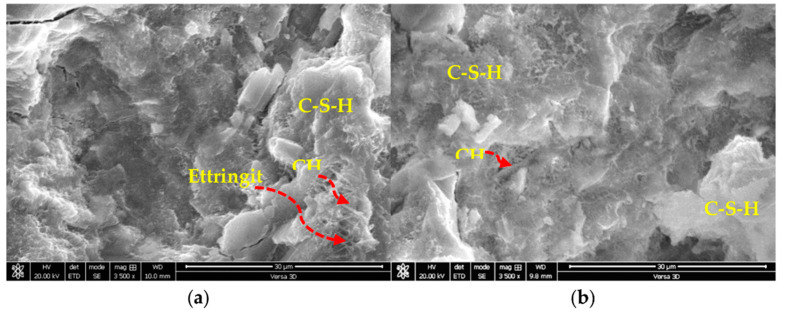

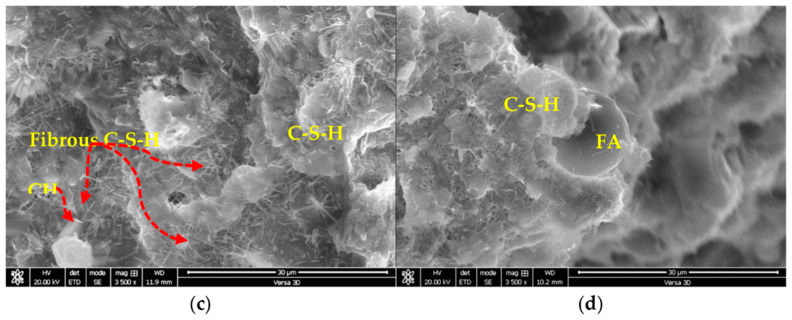
SEM images of concrete mixes (**a**) CTRL, (**b**) NP1, (**c**) NP2 and (**d**) FA.

The microstructural analysis of concrete mixes (CTRL, NP1, NP2, and FA) at a curing age of 56 days is shown in Figure 20. The microstructural analysis revealed the actual state of pozzolanic reactivity of each powder and its filling effect. In the control mix, ettringite needles and portlandite hexagonal crystals can be observed. The microstructure of NP1 mix shows similar features to control mix as few micropores are detected. The microstructure of NP2 confirms the abundant formation of C-S-H fibrous and regular structures in addition to the presence of micropores. The pozzolanic reactivity of NP1 and NP2 particles can be estimated from the compactness of their structures. On the other hand, the presence of unreacted FA particles in the FA mix is revealed by microstructural analysis. The intact form of FA was attributed to their latent pozzolanic reactivity, i.e., slow pozzolanic reactivity. The hydration products of the pozzolanic reaction are confirmed to be more reactive than NP1 in the NP2 mix.

## 4. Conclusions

Scoria rocks from various sources have varying physicochemical properties. The nature of pozzolanic reactivity is determined by the curing medium. The following can be concluded:

1. High-pressure steam curing (autoclave) is highly effective with materials containing tectosilicate minerals, namely 3D-framework structures, which convert to tobermorite under such conditions. The materials with single-chain silicate structures, on the other hand, did not generate tobermorite alone, which forms an enhanced type of tobermorite crystals in the presence of crystalline GFS.
2. Under low-pressure steam curing of NP and CH, the estimated amounts of the remaining CH in the NP1 and NP2 were about 7.1 and 5.1%, respectively, which means that 72 and 80% of portlandite, respectively, is consumed in the pozzolanic reaction.
3. In normal concrete mixes, the control mix (CTRL) has the highest peak value of the temperature profiles, followed by the NP2 mix, which has a substantial temperature gap when compared to the inert GFS mix. This temperature behavior was attributed to NP2’s pozzolanic reactivity.
4. When compared to the control concrete mix, the strength activity indices of NP1, NP2, and class F fly ash in their normal concrete mixes reached 74.3%, 82%, and 73.7%, respectively, after 56 days of normal curing conditions.

In general, NP with pyroxene minerals (single-chain silicates) is recommended for use in the normal concrete mixes, while NP with plagioclase feldspars minerals (3D-silicate structures) is recommended for autoclave curing. Pyroxene minerals with ground crystalline silica are highly recommended for use in autoclave curing. Precast and normal concrete technology will benefit from these findings.

## Figures and Tables

**Table 1:** Mix proportion of tested normal concrete mixes to assess pozzolanicity (kg/m^3^).

PC	NS	CS	CA10	CA20	Water
350	510	270	315	735	175

## Data Availability

All data, models, and code generated or used during the study appear in the submitted article.
